# ﻿Relationships among *Calibrachoa*, *Fabiana* and *Petunia* (Petunieae tribe, Solanaceae) and a new generic placement of Argentinean endemic *Petuniapatagonica*

**DOI:** 10.3897/phytokeys.194.68404

**Published:** 2022-04-15

**Authors:** Alejandrina Alaria, John H. Chau, Richard G. Olmstead, Iris E. Peralta

**Affiliations:** 1 Agronomy Faculty, National University of Cuyo, Alte. Brown 500, Luján de Cuyo, Mendoza, Argentina National University of Cuyo Mendoza Argentina; 2 Department of Biology, University of Washington, Seattle, Washington, 98195, USA University of Washington Seattle United States of America; 3 Centre for Ecological Genomics and Wildlife Conservation, Department of Zoology, University of Johannesburg, Auckland Park 2006, South Africa University of Johannesburg Johannesburg South Africa; 4 IADIZA CCT CONICET, Adrián Ruiz Leal s/n Parque general San Martín, Mendoza, Argentina IADIZA CCT CONICET Mendoza Argentina

**Keywords:** *Fabianaaustralis*, Patagonia, *Petuniapatagonica*, Petunieae, phylogeny

## Abstract

*Calibrachoa* Cerv., *Fabiana* Ruiz & Pav., and *Petunia* Juss. form a clade within tribe Petunieae (Solanaceae). Phylogenetic studies of Petunieae, either as part of a family-wide analysis or focusing on the genera *Calibrachoa* and *Petunia*, have either left *Fabiana* unsampled or included only a single species. These studies have found conflicting relationships among the three genera with all three possible topologies obtained. *Petuniapatagonica* (Speg.) Millán, originally described in the genus *Nierembergia* Ruiz & Pav., is morphologically distinct within *Petunia* and geographically disjunct from other members of the genus. For the first time, in this study we include multiple species of *Fabiana*, *Calibrachoa*, and *Petunia*, including *P.patagonica*. Using three chloroplast DNA regions and the nuclear gene GBSSI, or “waxy,” our results provide strong support for a sister group relationship between *Calibrachoa* and *Fabiana* and for the placement of *P.patagonica* within *Fabiana*. Since there is already a species *Fabianapatagonica* Speg., we provide the new name *Fabianaaustralis* Alaria nom. nov. to replace *Petuniapatagonica*.

## ﻿Introduction

Solanaceae Juss. are one of the most important families among Angiosperms not only for their fundamental contribution to the human diet but also for their diversity and ecological functions in many ecosystems wordwide, especially in the Neotropics (Olmstead 2013). Solanaceae are a family of approximately 90 genera and ca. 2700–2800 species (Olmstead and Bohs 2007; Olmstead et al. 2008, but see Knapp et al. 2004 for a higher estimate). Molecular phylogenetic studies in Solanaceae, initially based on chloroplast DNA sequence data (Olmstead and Palmer 1992; Spooner et al. 1993; Olmstead and Sweere 1994; Olmstead et al. 1999; Gemeinholzer and Wink 2001; Santiago-Valentin and Olmstead 2003; Clarkson et al. 2004; Bohs 2005; Levin et al. 2005, 2006; Weese and Bohs 2007; Olmstead et al. 2008), and more recently using a combination of chloroplast and nuclear loci and with nearly all genera and over 1,000 species sampled, have produced a robust dated molecular phylogeny (Goldberg et al. 2010; Särkinen et al. 2013; Ng and Smith 2015; Dupin et al. 2016).

Molecular phylogenetic studies unraveled relationships that were not consistent with traditional classification of the family and split up tribe Nicotianeae and resurrected tribe Petunieae to include most of the former Nicotianeae, excluding *Nicotiana* L., but including *Brunfelsia* L. (Olmstead et al. 1999, 2008; Olmstead and Bohs 2007). These results showed that the genera *Petunia* Juss., *Calibrachoa* Cerv., and *Fabiana* Ruiz & Pav. form a strongly supported clade and provided weak evidence suggesting that *Fabiana* is sister to *Calibrachoa* and that together they are sister to *Petunia*. The tribe Petuniae has been subject to several phylogenetic studies, with most of the focus on *Petunia* and *Calibrachoa* (Ando et al. 2005; Kulcheski et al. 2006; Stehmann et al. 2009; Fregonezi et al. 2012, 2013; Reck-Kortmann et al. 2014, 2015; Mäder and Freitas 2019). Few of these studies have included representatives of *Fabiana*, and when they do, relationships among the three genera sometimes do not agree (e.g., Särkinen et al. 2013; Reck-Kortmann et al. 2015; Mäder and Freitas 2019). Also, the rare species *Petuniapatagonica* (Speg.) Millán, which is morphologically distinct and geographically disjunct from other *Petunia* species, has an uncertain placement in Petunieae (Stehmann and Greppi 2013; Reck-Kortmann et al. 2015).

*Fabiana* is endemic to South America, distributed in southern Peru, Bolivia, Chile, and Argentina, with 15 species of shrubs adapted to the high Andean deserts of Puna, Prepuna, Monte, and Patagonia, growing from sea level to 4900 m elevation in sandy, rocky soils of very low fertility, low organic matter and variable salt content (Barboza and Hunziker 1993; Alaria and Peralta 2013; Alaria 2015). *Fabiana* species have characteristic morphological adaptations: leaflessness or small leaves, photosynthetic and resinous stems, and flat or cushion growth habits (Alaria 2015), and they play an important ecological role as codominant shrubs in some plant communities. *Petuniapatagonica* is restricted to the Patagonian region of Argentina, and its identity is controversial; it was first described in *Nierembergia* Ruiz & Pav. by Spegazzini (1897) and subsequently transferred to *Petunia* by Millán (1941).

In this study we explore the relationships among these three genera using sequences of the plastid *trnS*-*trnG*, *trnL*-*trnF* and *psbA*-*trnH* regions and partial sequences of the nuclear GBSSI or “waxy” gene, providing the first evidence of relationships among species of *Fabiana* and resolving the systematic position of the enigmatic species *Petuniapatagonica*.

## ﻿Materials and methods

### ﻿Taxon sampling

Leaf samples were obtained from fresh material collected in the natural habitat of species or from cultivated plants, and preserved in silica gel. Data, including collecting site, voucher, and herbarium where the voucher has been deposited, are indicated in Appendix 1 for specimens of tribe Petunieae: *Bouchetia* Dunal (1 species), *Brunfelsia* (1), *Calibrachoa* (4), *Fabiana* (9), *Nierembergia* (1), and *Petunia* (8, including *P.patagonica*), and outgroup taxa in the genera *Benthamiella* Speg. (1 species), *Nicotiana* (3), *Pantacantha* Speg. (1), and *Solanum* L. (1).

Additional specimens were analyzed for morphological traits, mainly species of *Calibrachoa*, *Fabiana*, *Nierembergia*, and *Petunia* in the Argentinean herbaria: BAB, CORD, CTES, LP, LIL, MEN, MERL, and SI, as well as in herbaria in Bolivia: LPB and HSB; Chile: LS; Perú: USM; and England: K (all acronyms are in accordance to *Index Herbariorum*; http://sweetgum.nybg.org/science/ih/). Specimens of *Petuniapatagonica* were examined and are cited after the species description in the Discussion.

### ﻿DNA amplification and sequencing

DNA extraction was performed using the Qiagen DNeasy Plant Mini Kit (http://www.qiagen.com). Three regions of chloroplast DNA were amplified. For the *trnL*-*trnF* region, the primers and amplification conditions of Taberlet et al. (1991) were used. This region was included in the phylogenetic analysis of Olmstead et al. (2008), where it provided essential characters for their classification of Solanaceae. Two other plastid fragments were selected for being more variable molecular markers in *Calibrachoa* (Fregonezi et al. 2012) and *Petunia* (Lorenz-Lemke et al. 2006; Kulcheski et al. 2006): the *trnS*-*trnG* region and the *psbA*-*trnH* region. For the *trnS*-*trnG* region, the primers and amplification conditions of Hamilton (1999) were used, and for the *psbA*-*trnH* region, the primers and amplification conditions of Sang et al. (1997) were used. The nuclear gene GBSSI or “waxy”, which has a single copy in tomato (*Solanumlycopersicum* L.) and diploid potato varieties (*Solanumtuberosum* L.) (van der Leij et al. 1991), was selected because it is an informative marker for phylogenetic analyses in Solanaceae (Peralta and Spooner 2001; Walsh and Hoot 2001; Levin and Miller 2005; Yuan et al. 2006; Spooner et al. 2008; Levin et al. 2011; Särkinen et al. 2013). The first waxy sequences of *Fabiana* specimens were obtained with primers initially designed for *Solanumlycopersicum* (Peralta and Spooner 2001), but new Petunieae-specific primers were generated: WAXY 5´ GTGGGTACTGAGGTTGGTCCTT and WAXY 3´ GGGCTCACTGTAACCACCCTAT, improving amplification of representative samples. Tomato and potato specimens were also amplified for waxy as controls for the expected fragment length, using a known molecular mass marker (Lambda/EcoRI-HindIII Marker). Purified PCR products were sequenced using standard Sanger sequencing methods at the Biogenomics Unit of the Institute of Biotechnology of the National Institute of Agricultural Technology (INTA Castelar), Buenos Aires, Argentina.

### ﻿Editing and aligning of chloroplast and nuclear sequences

The sequences were manually edited with PROcessor of SEQuences (PROSEQ) version 2.9 (Filatov 2002). Alignment was performed in BioEdit version 5.0.6. (Hall 2004), first using ClustalW and then adjusted by manual alignment. Waxy alignments were made by comparing sequences with the gene in *Solanumtuberosum* (GenBank accession X83220). Chloroplast fragments were aligned with *Petuniaaxillaris* sequences for *trnS*-*trnG* (JF918370), *trnL*-*trnF* (AY098702), and *psbA*-*trnH* (DQ225610).

### ﻿Phylogenetic analysis

We created three datasets for phylogenetic analyses. One dataset consisted of the three plastid loci for 21 species. A second dataset comprising 20 samples consisted of the nuclear waxy sequences. The third dataset comprising 18 samples consisted of the three plastid loci and the nuclear waxy gene concatenated for each species for which sequences for waxy and at least two of the three plastid loci were available. For each locus, we compared nucleotide substitution models using the Akaike Information Criterion from analyses in jModeltest 2.1.4 (Guindon and Gascuel 2003; Darriba et al. 2012) and chose an appropriate model within the 95% confidence interval. Phylogenetic analyses were performed using two different inference methods, maximum likelihood (ML) and Bayesian. In analyses with a concatenated sequence dataset, each locus was treated as a separate partition, and the GTR + gamma model of nucleotide substitution was used for each partition. In analyses with just waxy, the HKY + gamma model was chosen. We performed ML analyses using GARLI 2.0 (Zwickl 2006). We executed four replicates of each full search, and used a generation threshold for termination of 20,000 and score threshold for termination of 0.001. Default settings were used for all other parameters. We additionally performed bootstrap searches using a generation threshold for termination of 10,000. For concatenated datasets, 500 bootstrap replicates were done, and for the waxy dataset, 1,000 bootstrap replicates were done. Bayesian analyses were done in MrBayes 3.2.1 (Ronquist et al. 2012) with two runs with four chains each. For concatenated datasets, analyses were run for 10,000,000 generations, sampling every 1,000 generations. For the waxy dataset, analyses were run for 5,000,000 generations with a sampling frequency of 500 generations. We determined that convergence was attained when the average standard deviation of split frequencies was <0.05 and the estimated sample size of parameters was >200 in Tracer 1.5 (Rambaut and Drummond 2009). Majority-rule consensus trees were constructed after discarding the initial 25% of samples as burn-in.

## ﻿Results

**Figure 1.**
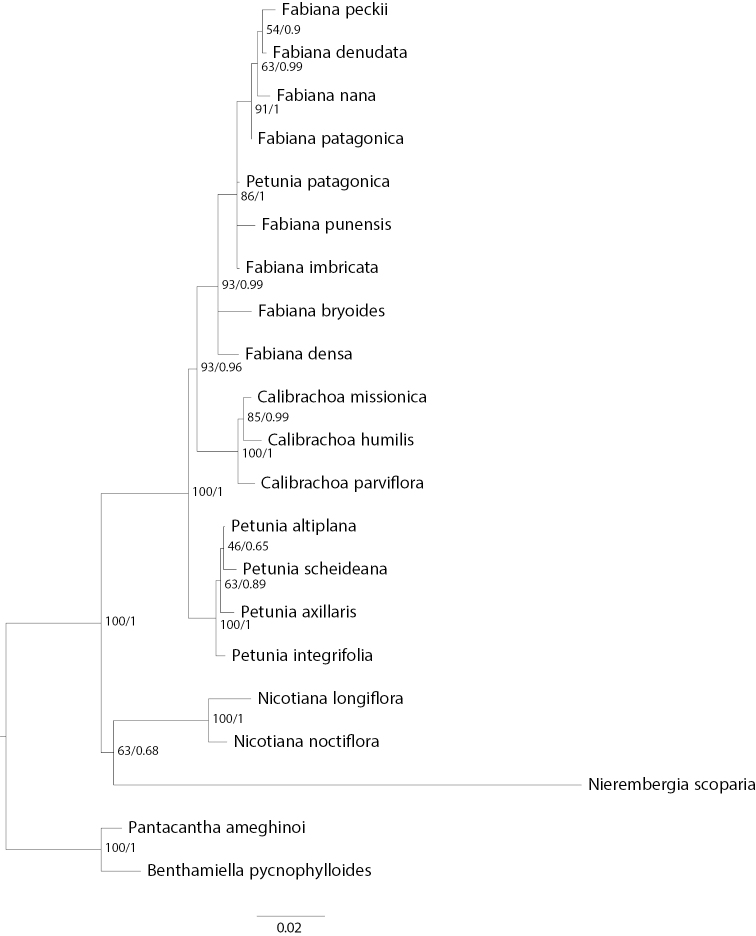
Chloroplast DNA tree of 17 Petunieae species and four outgroups. Phylogeny based on maximum likelihood analysis of concatenated *trnS*-*trnG*, *trnL*-*trnF*, and *psbA*-*trnH* chloroplast fragments. Maximum likelihood bootstrap values and Bayesian posterior probabilities shown at nodes.

The analyses of concatenated chloroplast sequences (Fig. 1) confirmed monophyly of the genera *Calibrachoa*, *Fabiana*, and *Petunia*, with the exclusion of *P.patagonica* (see below) and the phylogenetic relationships among the three genera was the same found by Olmstead et al. (2008) with *Petunia* sister to *Fabiana* and *Calibrachoa*, and the last two sister to each other. With limited sampling in the rest of tribe Petunieae and outgroups, *Nierembergia* is weakly supported as sister to *Nicotiana*. The analysis also strongly supports the placement of *Petuniapatagonica* within *Fabiana*.

**Figure 2.**
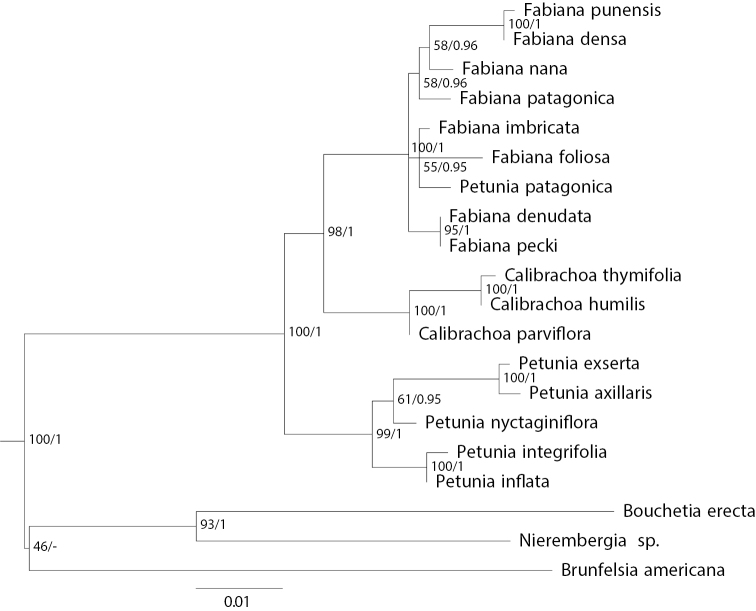
Nuclear waxy tree of 20 Petunieae species. Phylogeny based on maximum likelihood analysis of the nuclear waxy gene. Maximum likelihood bootstrap values and Bayesian posterior probabilities shown at nodes.

Waxy amplifications always showed a single band for a fragment of similar size to the one in potato and tomato controls. Although waxy gene copy number is unknown in *Fabiana*, *Calibrachoa*, and *Petunia*, it is expected to be a single copy and orthologous in the analyzed taxa, as has been demonstrated in other diploid Solanaceae species (van der Leij et al. 1991). The waxy phylogeny of 20 taxa (Fig. 2) is consistent with the chloroplast DNA analyses in recovering the monophyly of *Calibrachoa*, *Fabiana*, and *Petunia*, and the same relationships among them. Similarly, *Petuniapatagonica* is included within *Fabiana*. With strong support, *Nierembergia* is resolved as sister to *Bouchetia*.

**Figure 3.**
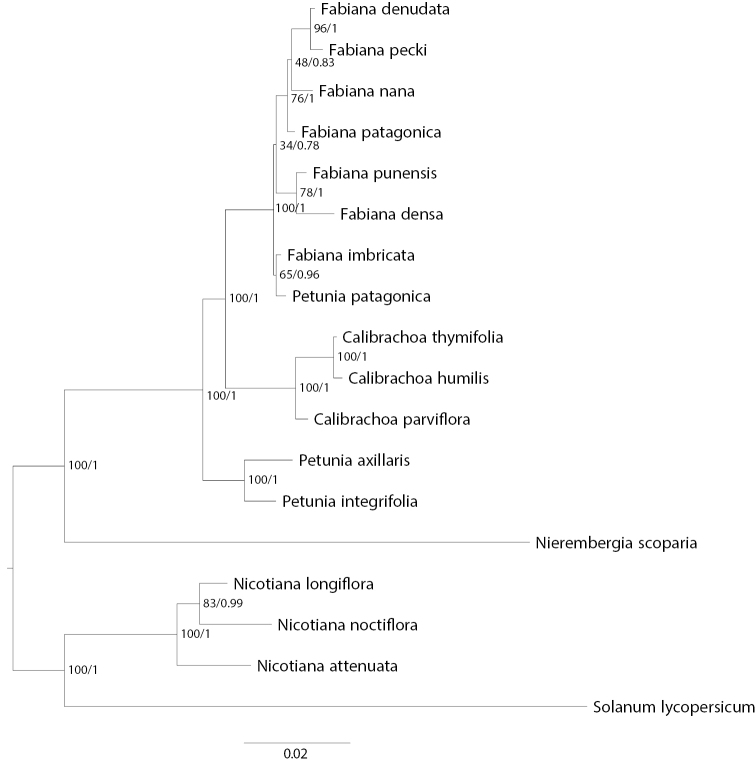
Combined chloroplast and nuclear DNA tree of 14 Petunieae species and four outgroups. Phylogeny based on maximum likelihood analysis of chloroplast fragments *trnS*-*trnG*, *trnL*-*trnF* and *psbA*-*trnH* and the nuclear waxy gene concatenated. Maximum likelihood bootstrap values and Bayesian posterior probabilities shown at nodes.

The results of the analyses of the concatenated sequences of chloroplast loci and waxy combined also recovered the same major relationships (Fig. 3). Monophyly of *Fabiana* with *Petuniapatagonica* nested within it is strongly supported (posterior probability = 1.0). In these analyses, *P.patagonica* is weakly supported as sister to *Fabianaimbricata* Ruiz and Pav. Additionally, *Fabiana* is resolved as sister to *Calibrachoa* with strong support, and *Petunia* is sister to the clade comprising *Fabiana* and *Calibrachoa*. *Nierembergia* forms a clade with the remaining representatives of Petunieae with strong support.

## ﻿Discussion

Sequences of three chloroplast markers and the nuclear gene waxy were informative for the inference of phylogenetic relationships among *Calibrachoa*, *Fabiana*, and *Petunia* in tribe Petunieae. Both the analyses of the combined chloroplast regions and the nuclear gene waxy corroborated *Calibrachoa* as sister to *Fabiana* with strong support, as obtained by Olmstead et al. (2008). A few studies of Petunieae with a single species of *Fabiana* sampled obtained results with *Fabiana* either sister to *Petunia* plus *Calibrachoa* (Särkinen et al. 2013) or *Petunia* (Reck-Kortmann et al. 2015; Mäder and Freitas 2019), in contrast to our results. All of our analyses also found *Petuniapatagonica* to be nested within *Fabiana*. This result is consistent with that of Reck-Kortmann et al. (2015), who found *P.patagonica* sister to *F.imbricata* and the combined clade sister to the rest of *Petunia*; but with only a single representative of *Fabiana*, that study was not able to reveal conclusively the placement of *P.patagonica* within *Fabiana*. Considering these phylogenetic results as well as shared morphological characteristics, geographical distribution, and chromosomal number between *Fabiana* and *Petuniapatagonica* (Reck-Kortmann et al. 2015), the transfer of *Petuniapatagonica* to the genus *Fabiana* is strongly supported.

### ﻿Circumscription of *Fabiana* and transfer of *Petuniapatagonica* to *Fabiana*

This study resolves the phylogenetic position of *Petuniapatagonica*, an enigmatic species of controversial generic affinity. Spegazzini (1897) originally described this species in the genus *Nierembergia* based on a specimen collected in Gulf Saint George, Argentina. The corolla and androecium resemble other *Nierembergia* species, with a narrow base to the corolla tube that expands distally. Subsequently, in a review of *Nierembergia*, Millán (1941) transferred the species to *Petunia*, considering its floral characters as closer to this genus. The species has several characteristics that differentiate it from all other *Petunia* species and has been considered an outlier in the genus based on its geographic distribution and chromosome number (Stehmann and Greppi 2013; Reck-Kortmann et al. 2015). The species was included in a molecular phylogenetic study with several species of *Petunia*, two species of *Calibrachoa*, but only one species of *Fabiana* and was found in a small clade with *F.imbricata*, which was sister to a clade comprising the other species of *Petunia* (Reck-Kortmann et al. 2015).

**Figure 4.**
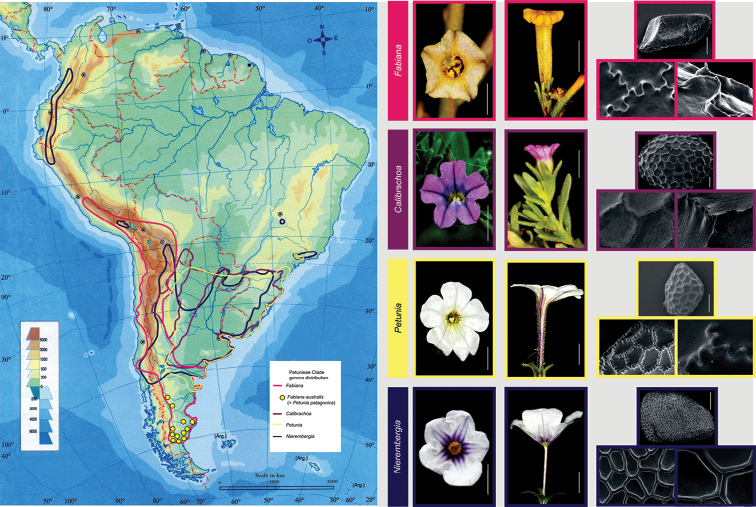
Geographic distribution of the genera *Fabiana*, *Calibrachoa*, *Petunia*, and *Nierembregia*, and the species *Fabianaaustralis* Alaria. Flowers and seeds of representative species: *Fabianapatagonica* Speg (first flower scale: 2.5 mm, second flower scale: 5 mm, seed scale: 0.5 mm, surface details magnifications 600× and 1,500×); *Calibrachoaparviflora* (Juss.) D’Arcy (first flower scale: 2.5 mm, second flower scale: 5 mm, seed scale: 0.5mm, surface details magnifications 500× and 1,500×); *Petuniaaxillaris* (Lam.) Britton, Sterns & Poggenb. (first flower scale= 5mm, second flower scale: 10mm, seed scale: 0.5mm, surface details magnifications 600× and 1,500×); *Nierembergiapulchella* Gillies ex Miers (first flower scale: 5mm, second flower scale: 10mm, seed scale: 0.5mm, surface details magnifications 600× and 1,500×). Photograph IBODA, Flora Argentina database.

Traditional classifications of *Petuniapatagonica* have relied primarily on morphology, but with the insight gained from molecular phylogenetic studies, we can see that taxonomists weighed different floral traits in assigning the species first to *Nierembergia* and then to *Petunia*, while overlooking the similarities with *Fabiana*, including the resinous stems and dorsifixed anthers. Other characteristics, such as the chromosome number of *Petuniapatagonica* (n = 9), match those found in *Fabiana* species (Acosta et al. 2006). The particular distribution of this species in southern Patagonia (Fig. 4) and the environment where it grows, are similar to those of other *Fabiana* species (e.g., *F.imbricata*, *F.foliosa* (Speg.) S.C.Arroyo *and F.nana* (Speg.) S.C.Arroyo). The results of the molecular phylogenetic analyses obtained in this work also support its transfer to the genus *Fabiana*.

#### ﻿ Fabiana australis

Taxon classificationPlantaeSolanalesSolanaceae

Alaria nom. nov.

A69084F1-6D0C-586A-B3BC-E646C78DF656

urn:lsid:ipni.org:names:77297063-1

Figures 4 , 5

Petunia patagonica (Speg.) Millán. Darwiniana 5: 544 1941.

##### Basionym.

*Nierembergiapatagonica* Speg. Revista Fac. Agron. Univ. Nac. La Plata 3: 357. 1897, non *Fabianapatagonica* Speg. (1897). Type: Argentina. Prov. Santa Cruz, Golfo de San Jorge, C. Ameghino, Febr. 1896, “in campis aridis glariosis” (holotype: LP 006658!).

##### Description.

Densely branched shrubs forming compact cushions of approximately 50 cm tall and up to 2.5 (-4) m in diameter; stems erect, leafy, glandular-pubescent, resinous. Leaves alternate but apparently verticillated by shortening of the internodes, sessile, fleshy, glandular; linear, elliptical or obovate, blade 3–4 (5) mm long by 1–2 mm wide. Flowers terminal, solitary, erect; flowering pedicels of 4–6 mm. Calyx tubular or slightly campanulate, (8–) 11–12 mm long, externally with dense glandulous indumentum, internally with scattered glandulous pubescence, short and broadly triangular lobes, 2–3 mm long by 2–3.5 mm wide, almost as long as wide. Corolla yellow with marked violet nerves, but also with color variation from light purple to deep violet, infundibuliform, 20–25 (–30) mm long, externally glandular, broad triangular lobes 2.5–5 mm long by 6–10 mm wide. Stamens heterodynamous, adhered to the middle third of the corolla, about 9 mm from the base of the corolla; filaments 2 short and 3 long, somewhat geniculate at the point of insertion with short glandular hairs scattered at the base; ellipsoid anthers of 1–1.5 mm. Ovary obovoid, 2.5–3 mm long, 1.2–1.5 mm wide, with nectariferous disc surrounding the base, 10–15 mm long style, truncated stigma, shallowly split. Capsule ovoid, 5–9 mm long by 3–5.5 mm wide. Seeds numerous, polyhedrical in shape, 2–2.5 mm long.

**Figure 5.**
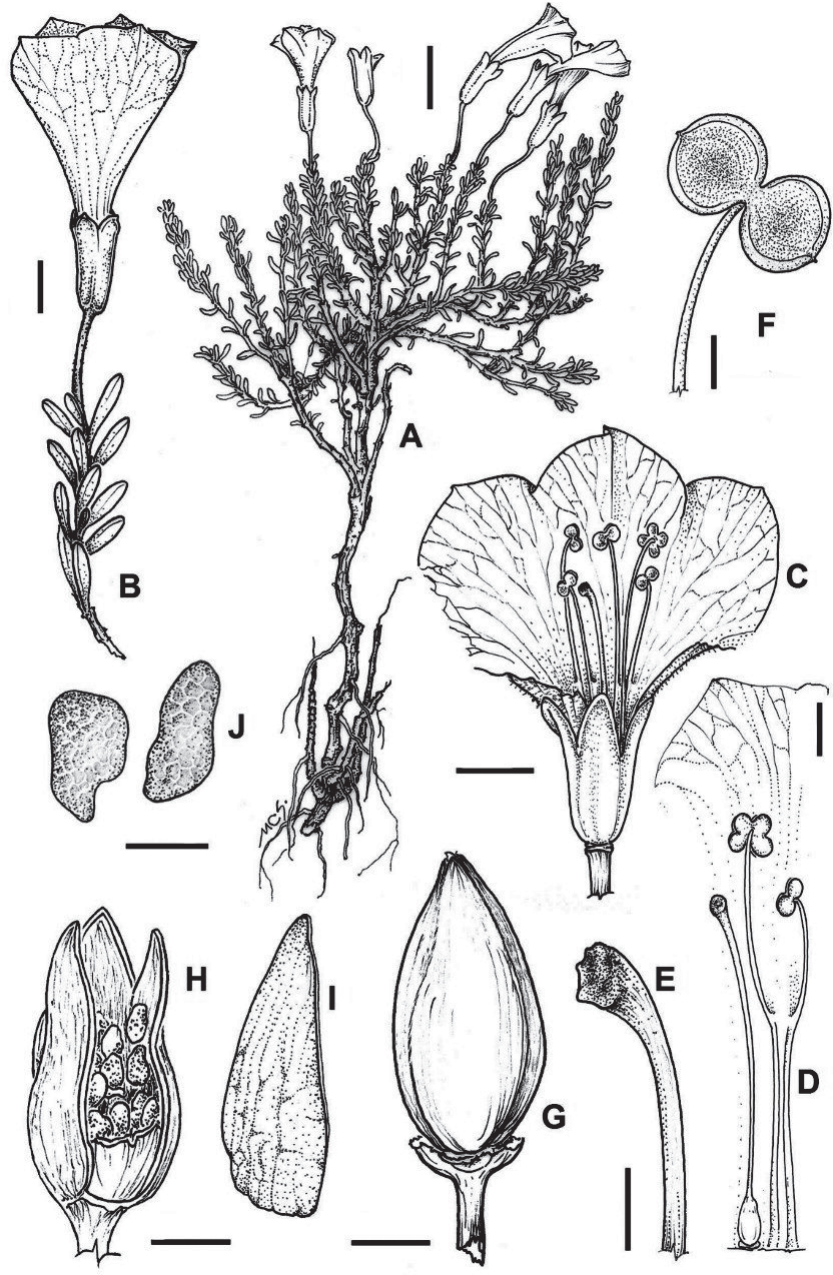
*Fabianaaustralis* Alaria. **A** plant **B** flowering branch **C** flower **D** corolla deployed showing gynoecium and stamens of different length **E** stigma **F** anthers **G** capsule **H** capsule showing seeds, **I** capsule valve **J** seeds. Scale bars: 10 mm (**A**); 4 mm (**B**); 5 mm (**C**); 2.5 mm (**D**); 1 mm (**E**); 0.5 mm (**F**); 2 mm (**G, H**). Illustration by Cecilia Scoones.

##### Common name.

“Mogote” meaning mound shape (Santa Cruz: Arroyo 1999)

##### Geographical distribution and habitat.

**Figure 6.**
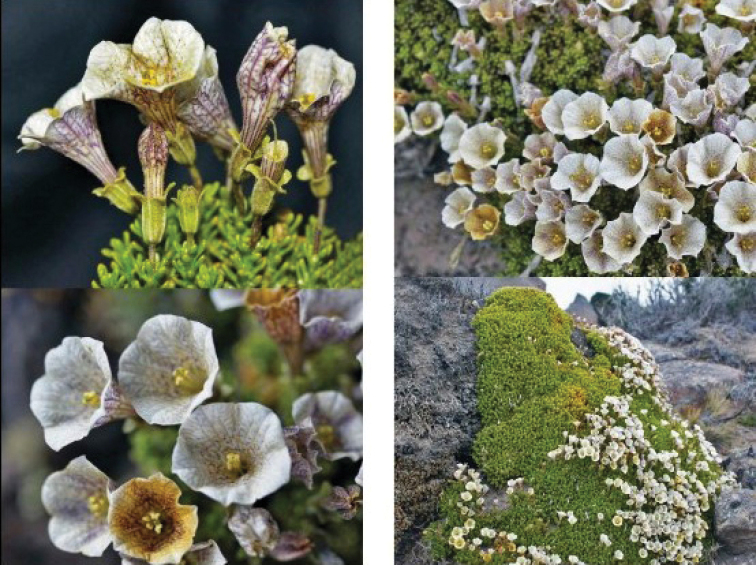
*Fabianaaustralis* Alaria. Plants habit and flower details (Zuloaga FO 13991, SI). Photograph IBODA, Flora Argentina database.

Endemic to Patagonian Argentina, in the provinces of Chubut and Santa Cruz, from 90 to 700 m elevation, inhabiting dry and cold environments, on stony, sandy soils, sometimes rich in silt and clay, poor in organic matter. It forms large populations of cushion shrubs with numerous showy flowers (Fig. 6).

##### Taxonomic notes.

The epithet *autralis* was selected based on the restricted distribution of this species in southern Argentina. It is not possible to use *patagonica* as a specific epithet in *Fabiana*, because it is already in use in *Fabianapatagonica* Speg. *Fabianaaustralis* is one of the southernmost species of the genus. It shares with *F.foliosa* and *F.nana* a similar habit forming dense cushions in dry, cold, and poor soils of Patagonian Argentina.

##### Representative specimens examined.

Argentina: **Santa Cruz**: Dpto. Corpen Aike, G.E. Barboza 3706 (CORD); O. Boelcke 16264 (BAB); A.A. Cocucci 3684 & 3723 (CORD); M.N. Correa 6527 (BAB); R.H. Fortunato 7492 (BAB); C.A. O’Donell 3794 (CORD00015699). Dpto. Deseado, L.M. Bernardello & M.R. Figueroa Romero 335 (CORD00015696!); O. Boelcke, 12214 (BAB); A.A. Cocucci 4175 (CORD); M.N. Correa 2644 & 6697 (BAB); B.E Leuenberger 4100 (B: D-14191 Berlin); M.C. Romanczuk 989 (UEC); F.B. Vervoorst 5658 (CORD 00015700!). Dpto. Güer Aike, A. Soriano 5062 (BAB). Dpto. Lago Argentino, G.E. Barboza 3732 (CORD); A.A. Cocucci 471 (CORD 00015694!); R.H. Fortunato 4967 (BAB, ARIZ, NY, HRP); C. Guerrido 785 (SI). Dpto. Magallanes, G.E. Barboza 3704 (CORD); O. Boelcke 15394 (BAB); Iter Patagonicum 762 L. Hauman & C.M. Hicken (SI); B.E. Leuenberger & S. Arroyo 3710 (CORD 00015698!). Dpto. Río Chico, M.N. Correa & E.G. Nicora 3517 (BAB; CORD 00015697!); G.E. Barboza 3696 (CORD); G.E. Barboza 3746 (CORD; SI 063988!); G.E. Barboza 3748 (CORD); O. Boelcke 12810 (BAB); J.M. Bonifacino 2986 (SI); C.M. Hicken 10245 (SI); F.O. Zuloaga 13978 & 13991 (SI). Valle del Río Santa Cruz C. Burmeister s.n. & 95 (SI); M. Gentili 330 (BAB); J. Koslowsky 122 (CORD 00015695!). Without locality A. Donat 206 (SI); P.K.H. Dusén 5496 (SI); E. Molina Massey 31 (SI); Tessleff 5496 (SI). **Chubut**: Dpto. Futaleufú, A.A. Cocucci 3997 (CORD 00022097!); Dpto. Paso de Indios, S.C. Arroyo 208 (BAB, LIL, K); Dpto. Sarmiento, A. Alaria 321 (MERL). Dpto. Languiñeo, A. Alaria 324 (MEN).

Taxonomic characters differentiating *Calibrachoa*, *Fabiana*, *Petunia*, and *Nierembregia* are described in the following key. Geographic distribution of the four genera and *Petuniapatagonica*, as well as photographs of flowers and seeds of representative species of each genus, are illustrated in Figure 4.

### ﻿Key to genera

**Table:** 

1	Resinous shrubs to camephytes, stems densely leafy to partially foliated and even aphyllous; reduced membranaceous, slightly fleshy or leathery leaves. Dorsifixed anthers, usually elongated	***Fabiana***
–	Non resinous, annual or perennial herbs, rarely subshrubs; leafy stems, developed membranaceous to fleshy leaves. Ventrifixed anthers with different shapes: reniform, globose, or ovate	**2**
2	Hypocrateriform corolla with narrow and cylindrical tube. Androecium with 5 fertile stamens equal in length or heterodynamous, generally with 2 longer and 3 shorter stamens, adnate at the top edge of the corolla tube and generally connivent around the style; wide stigma usually tightly arranged between the anthers; staminal filaments and style apex usually straight. Nectary absent. Polyhedral seed, straight embryo	***Nierembergia***
–	Infundibuliform to campanulate, rarely hypocrateriform, corolla with wide tube. Androecium with 5 fertile heterodynamous stamens, generally with 2 longer, 2 medium length, and one shorter stamen, or 4 subequal and one shorter stamen, adnate at the top edge of the corolla tube but rarely connivent around the style; narrow stigma, staminal filaments and apex style usually curved. Nectary present. Ellipsoid, round, or reniform seed, straight or slightly curved embryo	**3**
3	Corolla with reciprocative aestivation, the induplicated anterior lobe covering the other four conduplicated lobes, or rarely imbricate aestivation; calyx usually divided nearly to the middle, lobes narrowing towards the apex; seed episperm with straight anticlinal cell walls	***Calibrachoa***
–	Corolla with imbricate aestivation; deeply lobed calyx, lobes linear or spatulate, widening towards the apex; seed episperm with wavy anticlinal cell walls	***Petunia***

## Supplementary Material

XML Treatment for Fabiana australis

## ﻿Appendix 1

Voucher and locality information for specimens of species collected for this study and GenBank accession numbers for sequences used, with those generated for this study in bold.

**Table A1.**

Species	Voucher	Location	Coordinates	*trnL*-*trnF*	*trnS*-*trnG*	*psbA*-*trnH*	waxy
**Petunieae Tribe**							
*Bouchetiaerect*a DC ex Dunal	D’Arcy 18213 MO	Mexico	–	–	–	–	**OK120263**
*Brunfelsiaamericana* L.	no voucher	Cultivated. USA, Matthaei Bot Gard	–	–	–	–	**OK166947**
*Calibrachoahumilis* (R.E.Fr.) Stehmann & Semir	no voucher	Cultivated. Argentina, FCA, UNCuyo	33°00'28.2"S, 68°52'16.4"W	**MZ855907**	**OK120228**	**MZ855925**	**OK120243**
*Calibrachoaparviflora* (Juss) D´Arcy	Alaria 432 MERL	Cultivated. Argentina, FCA, UNCuyo	33°00'28.2"S, 68°52'16.4"W	**MZ855908**	**OK120229**	**MZ855926**	**OK120244**
*Calibrachoamissionica* Stehmann & Semir	no voucher	Cultivated. Argentina, FCA, UNCuyo	33°00'28.2"S, 68°52'16.4"W	**MZ855909**	**OK120230**	**MZ855927**	–
*Calibrachoathymifolia* (A.St.-Hil.) Stehmann & Semir	no voucher	Cultivated. Argentina, FCA, UNCuyo	33°00'28.2"S, 68°52'16.4"W	**MZ855910**	–	**MZ855928**	**OK120245**
*Fabianabryoides* Phil.	Alaria 444 MERL	Argentina, Jujuy	22°31'47.8"S, 66°18'45.4"W	**MZ855911**	**OK120231**	**MZ855929**	**_____**
*Fabianadensa* J. Rémy	Alaria 365 MERL	Bolivia, Potosi	19°52'48.2"S, 65°40'44.4"W	**MZ855912**	**OK120232**	**MZ855930**	**OK120246**
*Fabianadenudata* Miers	Alaria 356 MERL	Argentina, Mendoza	32°29'16.3"S, 69°05'07.5"W	**MZ855913**	**OK120233**	**MZ855931**	**OK120247**
*Fabianafoliosa* (Speg.) S.C.Arroyo	Barboza 3760 CORD	Argentina, Santa Cruz	47°20'09"S, 70°59'05"W	–	–	**MZ855932**	**OK120248**
*Fabianaimbricata* Ruiz & Pav.	Alaria 397 MERL	Argentina, Mendoza	35°51'451"S, 69°48'27.5"W	**MZ855914**	**OK120234**	**MZ855933**	**OK120249**
*Fabiananana* (Speg.) S.C.Arroyo	Alaria 316 MERL	Argentina, Chubut	45°47'44.5"S, 69°04'56.7"W	**MZ855915**	**OK120235**	**MZ855934**	**OK120250**
*Fabianapatagonica* Speg.	Alaria 359 MERL	Argentina Jujuy	22°57'30"S, 65°25'39"W	**MZ855916**	**OK120236**	**MZ855935**	**OK120251**
*Fabianapeckii* Niederl.	Alaria 403 MERL	Argentina, Mendoza	34°31'55.2"S, 68°28'14.7"W	**MZ855917**	**OK120237**	**MZ855936**	**OK120252**
*Fabianapunensis* S.C.Arroyo	Alaria 048 MERL	Argentina, Tucumán	26°38'39.5"S, 65°49'12.5"W	**MZ855918**	**OK120238**	**MZ855937**	**OK120253**
*Nierembergiascoparia* Sendtn.	Alaria 431 MERL	Cultivated. Argentina, FCA, UNCuyo	33°00'28.2"S, 68°52'16.4"W	**MZ855920**	**OK120240**	**MZ855939**	**OK120255**
*Petuniaaltiplana* T. Ando & Hashim	–	–	–	AY772868	DQ792185	DQ791917	–
*Petuniaaxillaris* (Lam.) Britton, Sterns & Poggenb.	Alaria 430 MERL	Argentina, Mendoza	32°58'45.7"S, 68°58'8"W	AY098702	JF918370	DQ225610	**OK120258**
*Petuniaexserta* Stehmann	Chau 312 WTU	Cultivated. USA, University of Washington	–	–	–	–	**OK120259**
*Petuniainflata* R.E. Fr.	Olmstead S-62 WTU	Cultivated. USA, seed from Birmingham seed collection	–	–	–	–	**OK120260**
*Petuniaintegrifolia* (Hook.) Schinz & Tell.	Chau 311 WTU	Cultivated. USA, University of Washington	–	AY772873	JN565848	DQ208151	**OK120261**
*Petunianyctaginiflora* Juss.	Olmstead S-63 WTU	Cultivated. USA, seed from Birmingham seed collection	–	–	–	–	**OK120262**
*Petuniapatagonica* (Speg.) Millán	Alaria 321 MERL	Argentina, Chubut	45°56'16.8"S, 69°09'12.4"W	**MZ855919**	**OK120239**	**MZ855938**	**OK120254**
*Petuniascheideana* L.B. Sm. & Downs	–	–	–	AY772870	DQ792448	DQ792149	–
**Outgroups**							
*Benthamiellapycnophylloides* Speg	Barboza 3688 CORD	Argentina, Santa Cruz	46°57'2"S, 67°22'24.6"W	**MZ855921**	**OK120241**	**MZ855940**	–
*Nicotianaattenuata* Torr. Ex S. Watson	–	–	–	AY098697	AJ584953	MG182422	KR083023
*Nicotianalongiflora* Cav.	Alaria 437 MERL	Argentina, Mendoza	32°59'47.7"S, 68°56'1.5"W	**MZ855923**	AJ584951	**MZ855942**	**OK120256**
*Nicotiananoctiflora* Hook.	Alaria 438 MERL	Argentina, Mendoza	32°59'49.4"S, 68°55'56.6"W	**MZ855924**	AJ584975	GQ248352	**OK120257**
*Pantacanthaameghinoi* Speg.	Barboza 3775 CORD	Argentina, Neuquén	38°52'0"S, 70°34'36.2"W	**MZ855922**	**OK120242**	**MZ855941**	–
*Solanumlycopersicum* L.	–	–	–	KY887587	HQ856092	KY887587	DQ169036
